# Poly(ADP-ribose)polymerase 1 stimulates the AP-site cleavage activity of tyrosyl-DNA phosphodiesterase 1

**DOI:** 10.1042/BSR20140192

**Published:** 2015-07-29

**Authors:** Natalia A. Lebedeva, Rashid O. Anarbaev, Maria Sukhanova, Inna A. Vasil’eva, Nadejda I. Rechkunova, Olga I. Lavrik

**Affiliations:** * Institute of Chemical Biology and Fundamental Medicine, Lavrentiev av. 8, Novosibirsk 630090, Russia; † Department of Natural Sciences, Novosibirsk State University, 2 Pirogov Street, Novosibirsk 630090, Russia

**Keywords:** clustered damages, fluorescently-labelled proteins, poly(ADP-ribose)polymerase 1, protein–protein interaction, tyrosyl-DNA phosphodiesterase 1

## Abstract

The influence of poly(ADP-ribose)polymerase 1 (PARP1) on the apurinic/apyrimidinic (AP)-site cleavage activity of tyrosyl–DNA phosphodiesterase 1 (TDP1) and interaction of PARP1 and TDP1 were studied. The efficiency of single or clustered AP-site hydrolysis catalysed by TDP1 was estimated. It was shown that the efficiency of AP-site cleavage increases in the presence of an additional AP-site in the opposite DNA strand depending on its position. PARP1 stimulates TDP1; the stimulation effect was abolished in the presence of NAD^+^. The interaction of these two proteins was characterized quantitatively by measuring the dissociation constant for the TDP1–PARP1 complex using fluorescently-labelled proteins. The distance between the N-termini of the proteins within the complex was estimated using FRET. The data obtained suggest that PARP1 and TDP1 bind in an antiparallel orientation; the N-terminus of the former protein interacts with the C-terminal domain of the latter. The functional significance of PARP1 and TDP1 interaction in the process of DNA repair was demonstrated for the first time.

## Abstract (precis)

Poly(ADP-ribose)polymerase 1 (PARP1), the key DNA repair regulator, physically and functionally interacts with tyrosyl-DNA phosphodiesterase 1 (TDP1) and stimulates its abasic site cleaving activity. These interactions allow suggesting PARP1 contribution in the alternative Tdp1 related repair pathway.

## INTRODUCTION

Protein–protein interactions play a significant role in DNA repair processes and their regulation, which is very important in the case of the repair of clustered damages (or multiple lesions), where oxidized bases, apurinic/apyrimidinic (AP) sites and strand breaks are situated within one or two turns of the DNA helix and can be located in both the DNA strands. Such damages appear under the action of ionizing radiation and radiomimetic drugs and present a significant problem for the cell [1]. The repair of an individual damage within a cluster must be strongly regulated to prevent formation of double-strand breaks that are highly toxic for the cell [1]. AP sites are among the most frequent endogenous lesions in cellular DNA and their repair is critical for genome stability and cell survival. The presence of AP sites within clusters as well as breaks in the opposite DNA strand increase the probability of double-strand break formation compared with the case of multiple damages composed of oxidized bases [2].

One of the key mechanisms modulating the activity of DNA repair systems is protein poly(ADP-ribosyl)ation catalysed by poly(ADP-ribose)polymerase 1 (PARP1). Interacting with DNA breaks in the presence of NAD^+^, PARP1 displays enzymatic activity resulting in synthesis of the negatively-charged polymer, poly(ADP-ribose) (PAR). This polymer is covalently bound to PARP1 itself and to acceptor proteins [3]. The auto-modification of PARP1 is thought to result in its dissociation from DNA, promoting regulation of DNA repair. We have recently found that PARP1 interacts with AP sites via formation of a Schiff base and possesses weak AP- and 5′-deoxyribose phosphate (dRP)-lyase activities [4,5]. The data suggest that this protein is also involved in regulation of AP site processing within clustered damages.

Recently, we have shown that human tyrosyl–DNA phosphodiesterase 1 (TDP1) cleaves AP sites with the formation of the 3′- and 5′-phosphate termini, suggesting a novel AP-endonuclese independent pathway of AP-site processing [6–9] that can be involved in the repair of AP sites clustered with bulky DNA lesions [7]. The unique enzymatic activity of TDP1 removes the covalently-linked adducts of DNA topoisomerase I (Top1) from the DNA 3′-end [10–12]. TDP1 is now considered a general 3′-DNA end-processing enzyme that acts within the single-stranded break repair complex to remove adducts and to prepare the broken DNA ends bearing 3′-phosphate groups for further processing by repair enzymes [13]. Thus, TDP1 may function to remove a variety of adducts from 3′-DNA ends during DNA repair [14–16]. TDP1 was also detected in the complex with several BER (base excision repair) enzymes; among them was PARP1 [17]. TDP1 was recently shown to interact physically with and to be a target for modification by PARP1 [18]. However, it has so far not been revealed whether the interaction between PARP1 and TDP1 plays a functional role in AP site cleavage catalysed by TDP1 and interaction of these proteins has not been quantitatively characterized. In the present study, we demonstrate that PARP1 stimulates the AP-site cleavage activity of TDP1 in the repair of clustered damages and suggest that tight contact of these proteins is most probably important for the AP-site cleavage activity of TDP1 in the presence of PARP1.

## MATERIALS AND METHODS

### Materials

γ-[^32^P]ATP (5000 Ci/mmol) was produced in the Laboratory of Biotechnology (ICBFM). T4 polynucleotide kinase was purchased from Biosan; reagents for electrophoresis and buffer components were from Sigma. 5(6)-Carboxyfluorescein *N*-hydroxysuccinimide ester and 5-carboxy-tetramethylrhodamine *N*-hydroxysuccinimide ester were from Sigma.

Recombinant human TDP1 and PARP1 were expressed in the *Escherichia coli* system [strain BL21 DE3 (pLys E)] and purified as described previously ([7,19] and [20] respectively). The recombinant plasmid pET 16B-TDP1 was kindly provided by Dr K.W. Caldecott (University of Sussex, U.K.). The plasmid pET32 containing the human PARP1 gene was kindly provided by Dr M.S. Satoh (Laval University, Canada). The recombinant purified uracil–DNA glycosylase (UDG) was a generous gift from Dr S.N. Khodyreva (ICBFM).

The deoxyuridine monophosphate (dUMP)-containing 32-mer oligodeoxyribonucleotides were synthetized in the Laboratory of Medicinal Chemistry (ICBFM). The sequences of the oligonucleotides used in the experiments were as follows:

**(C)**
5′-GGAAGACCCTGACGTTUCCCAACTTAATCGCC-3′
3′-CCTTCTGGGACTGCAACGGGTTGAATTAGCGG-5′
**(13)**
5′-GGAAGACCCTGACGTTUCCCAACTTAATCGCC-3′
3′-CCTTCTGGGACTGCAACGGGTTGAATTAGUGG-5′
**(5)**
5′-GGAAGACCCTGACGTTUCCCAACTTAATCGCC-3′
3′-CCTTCTGGGACTGCAACGGGTUGAATTAGCGG-5′
**(4)**
5′-GGAAGACCCTGACGTTUCCCAACTTAATCGCC-3′
3′-CCTTCTGGGACTGCAACGGGUTGAATTAGCGG-5′
**(0)**
5′-GGAAGACCCTGACGTTUCCCAACTTAATCGCC-3′
3′-CCTTCTGGGACTGCAAUGGGTTGAATTAGCGG-5′
**(−3)**
5′-GGAAGACCCTGACGTTUCCCAACTTAATCGCC-3′
3′-CCTTCTGGGACTGUAACGGGTTGAATTAGCGG-5′
**(−5)**
5′-GGAAGACCCTGACGTTUCCCAACTTAATCGCC-3′
3′-CCTTCTGGGACUGCAACGGGTTGAATTAGCGG-5′
**(−14)**
5′-GGAAGACCCTGACGTTUCCCAACTTAATCGCC-3′
3′-CCUTCTGGGACTGCAACGGGTTGAATTAGCGG-5′,

where U designates the dUMP residue, which is converted into the AP site by the following treatment with UDG. The same set of the fluorescently-labelled oligonucleotides was synthetized and DNA duplexes with 5′-fluorescein (FAM) in the upper strand and 5′-rhodamine (ROX) in the bottom strand were obtained.

### Radioactive labelling of the oligonucleotides

The oligonucleotides were 5′-[^32^P]-labelled with T4 polynucleotide kinase and γ−[^32^P]ATP as described [21]. Unreacted γ−[^32^P]ATP was removed by passing the mixture over a MicroSpinTM G-25 column (Amersham) using the manufacturer′s suggested protocol. The complementary oligonucleotides were annealed in equimolar amounts by heating the solution to 95^º^C for 3 min, followed by slow cooling to room temperature.

### Endonuclease assays

The standard reaction mixtures (10 μl) contained 50 mM Tris/HCl (pH 7.5), 50 mM NaCl, 10 nM [^32^P]-labelled or 500 nM fluorescent DNA substrate and the necessary amounts of the proteins. For the preparation of the natural AP site, all DNA duplexes were first incubated in the reaction buffer with UDG (0.5 units/μl) for 15 min at 37°C. After addition of TDP1 or TDP1 and PARP1 simultaneously to final concentrations of 10 or 500 nM (for radioactive and fluorescent DNA respectively) the reaction mixtures were incubated at 37°C for 30 min. The reactions were then terminated by the addition of formamide dye and the mixtures were heated for 3 min at 90°C. The products were analysed by electrophoresis in 20% polyacrylamide gels with 8 M urea [21] and visualized using Molecular Imager (Bio-Rad) and Quantity One software.

### Protein binding to DNA

The protein–DNA complexes were analysed by the gel retardation assay (EMSA). The reaction mixture (10 μl) contained 50 mM Tris/HCl (pH 7.5), 50 mM NaCl, 10 nM 5′-[^32^P]-labelled DNA and TDP1 at various concentrations. The AP DNA duplex was first incubated in the reaction buffer with UDG (0.5 units/μl) for 15 min at 37°C. The complexes of TDP1 with DNA were formed on ice for 5 min. Then the loading buffer [1:5 (v/v)] containing 20% glycerol and 0.015% Bromophenol Blue was added to the sample. The protein–DNA complexes were electrophoresed under non-denaturing conditions. To separate the products of complex formed, 5% polyacrylamide gel (acrylamide–bis-acrylamide=60:1) was used; TBE was the electrode buffer. Electrophoresis was performed at 4^º^C with voltage decrease in 17 V/cm. The positions of the radioactively-labelled oligonucleotide and the protein–DNA complexes were determined autoradiographically using Molecular Imager (Bio-Rad).

### Labelling of the proteins with fluorescein and TAMRA

For protein labelling, we used 5(6)-carboxyfluorescein *N*-hydroxysuccinimide ester and 5-carboxy-tetramethylrhodamine *N*-succinimidyl ester, in which the carboxy groups were activated with N-hydroxysuccinimide (NHS). NHS ester is easily displaced by nucleophilic attack from primary amino groups of the N-terminal amino acid of a protein and lysine side chains at the water-accessible surface of a protein, thus forming an amide bond with the original carboxy group of fluorescein or tetramethyl-6-carboxyrhodamine (TAMRA). Succinimidyl esters are amine-reactive probes with strong dependence of the labelling reaction on the pH. In buffers with neutral pH, primary labelling of the terminal amino group can be achieved [22]. For labelling of TDP1 and PARP1, 2–3 μl of 1 mM NHS esters in DMSO was added to 1 ml of 2–10 μM proteins in 100 mM NaCl and 50 mM MES buffer (pH 8.0). The reaction mixtures were incubated for 8 h at 4°C and then filtered through the 300 kDa cut-off filter on Vivaspin ultrafiltration spin columns (Sartorius Stedim Biotech GmbH) to remove large protein aggregates. The conjugates were separated from the reagents by several steps of dilution in 100 mM NaCl, 2 mM DTT, 100 mM Tris/HCl buffer (pH 8.0) and following concentration by centrifugation on a Vivaspin ultrafiltration spin column (cut-off 30 kDa). After addition of glycerol to 50% (v/v), the final preparation was stored at −20°C. The concentration and stoichiometry of the fluorescein (FAM)–or TAMRA–protein conjugates was determined using the extinction coefficients: ε_280_=128000 cm^−1^·M^−1^ for TDP1, ε_280_=120000 cm^−1^·M^−1^ for PARP1, ε_280_=23400 cm^−1^·M^−1^ and ε_495_=78000 cm^−1^·M^−1^ for fluorescein and ε_280_=27300 cm^−1^·M^−1^ and ε_565_=91000 cm^−1^·M^−1^ for TAMRA.

### Fluorescence assays of PARP1 and TDP1 binding

Binding of the proteins was examined by fluorescence titration experiments. Fluorescence intensities of solutions of the FAM-labelled protein at a fixed concentration were measured in the absence and in the presence of various concentrations of the partner protein. The reaction mixtures (100 μl) contained 50 mM Tris/HCl (pH 8.0), 1 mM DTT, 50 mM NaCl, 20 nM PARP1–FAM (degree of labelling 30%) or TDP1–FAM (degree of labelling 40%) and 0–200 nM unlabelled TDP1 or PARP1. The mixtures were incubated at 25°C for 15 min.

To detect TDP1–PARP1 interaction by the FRET approach, the fluorescence intensity of the FAM-labelled PARP1 (donor) was measured in the absence and in the presence of various concentrations of the TAMRA-labelled TDP1 (acceptor). The reaction mixtures (100 μl) contained 50 mM Tris/HCl (pH 8.0), 1 mM DTT, 50 mM NaCl, 20 nM PARP1–FAM (degree of labelling 30%) and 0–480 nM TDP1–TAMRA (degree of labelling 38%). The mixtures were incubated at 25°C for 15 min.

The samples were prepared and measured in Corning™ black 96-well polypropylene assay plates. Fluorescence measurements and data analysis were performed using a POLARstar OPTIMA multifunctional microplate reader and MARS Data Analysis Software (BMG LABTECH GmbH). Excitation of the fluorescent probes was performed at 485 nm (485BP1 filter) with fluorescence detection at 520 nm (EM520 filter). The degree of binding (*D*_b_) was estimated by:

$$D_{b}=\frac{F-F_{0}}{F_{\max}-F_{0}}$$

where *F* is the fluorescence intensity of the PARP1–FAM (or TDP1–FAM) conjugate at different concentrations of TDP1 (or PARP1), *F*_0_ and *F*_max_ are the fluorescence intensities in the absence of TDP1 (or PARP1) and at saturated concentrations of these proteins respectively.

The dissociation constant (*K*_d_) was estimated by:

$$D_{b}=\frac{1}{1+\frac{K_{d}}{\left[C\right]}}$$

where [C] is the protein concentration.

## RESULTS AND DISCUSSION

**Figure 1:**
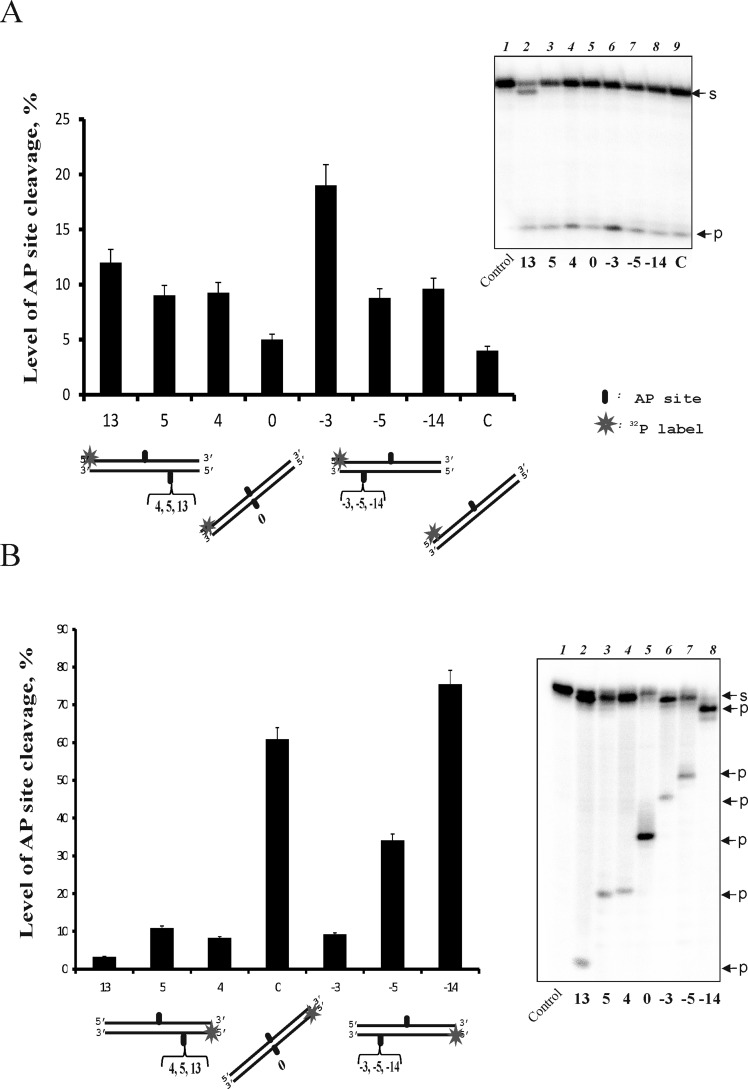
Efficiency of the AP site cleavage catalysed by TDP1 in the upper (A) and the bottom (B) DNA strands The DNA duplexes containing an AP site in the ^32^P-labelled strand and in defined positions of the opposite strand were treated by TDP1 (10 nM). Averages and experimental errors were taken from at least three experiments. Control on the denaturing polyacrylmide gel corresponds to AP DNA treated by UDG. S denotes the initial substrate, P denotes the products of hydrolysis.

We have shown earlier that TDP1 cleaves the AP site located opposite a bulky lesion more effectively compared with individual AP sites and that the cleavage efficiency depends on the AP site position in the DNA structure [7,9]. To further investigate a possible implication of TDP1 in AP site repair, particularly within clusters, we analysed the hydrolysis of the AP site by TDP1 in the middle of the labelled strand of 32-mer DNA duplexes that also contain AP sites in different positions of the opposite (unlabelled) strands (Figure 1A). It was clearly observed that the efficiency of AP site cleavage increased in the presence of the additional AP site in the opposite DNA strand depending on the position of this AP site. The minimal effect on the cleavage reaction was observed when two AP sites were located directly opposite each other (position 0 on the diagram). The strongest increase in the activity analysed (more than 4-fold) was observed in position 3 of the additional AP site in comparison with position 0.

We have also analysed TDP1-catalysed hydrolysis of the AP site located in the bottom DNA strand (Figure 1B). The efficiency of this reaction depends on the position of the AP site to a greater extent than does hydrolysis when the AP site is in the upper strand. Interestingly, the AP site in position 0 is cleaved significantly more effectively than the opposite site (~10-fold), whereas the AP site in position 3 is cleaved with low efficiency. The highest efficiency of TDP1 cleavage is observed for the AP site in position 14, probably due to its location near the 3′-end. It should be noted that not only AP-site cleaving, but also 3′-nucleosidase activity of TDP1 is sensitive to the position of the AP site in the bottom strand, being more intensive on this strand; in the case of the upper strand, the product corresponding to the single-nucleoside shortened strand is observed only for the DNA structure 13 (Figure 1A, lane 2).

**Figure 2:**
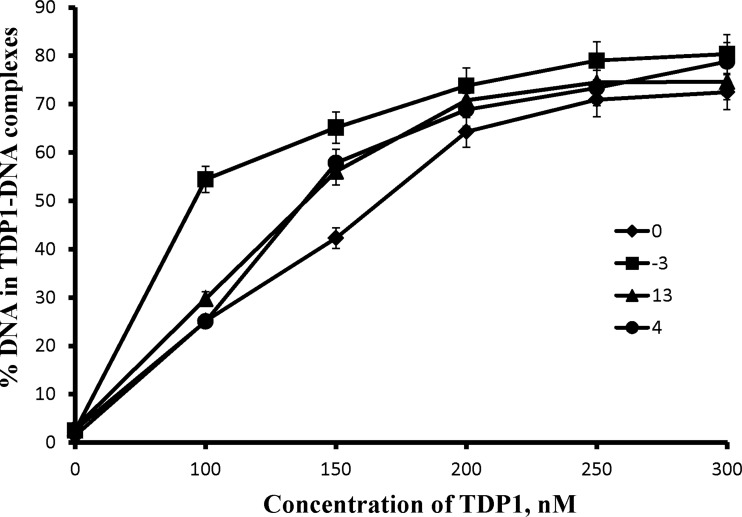
TDP1 binding to AP DNA structures The 5′-end labelled DNA substrates (10 nM) were incubated with increasing concentrations of TDP1 and subjected to native gel electrophoresis. The curves show quantitative analysis of the data from three independent experiments.

The EMSA data on TDP1 binding to the DNA structures (Figure 2) demonstrate that the efficiency of AP site cleavage in the upper strand correlates with the TDP1 affinity for the DNA. The AP site is known to induce a local distortion of the DNA duplex followed by its bending [23]. Therefore, the AP site cleavage efficiency for the AP site in the upper strand may reflect duplex destabilization due to an additional AP site in the opposite strand, whereas cleavage of an AP site in the bottom strand depends mainly on its position in this strand. It seems very possible that AP sites in the upper and in the bottom strands are processed by different TDP1 molecules. This ability is confirmed by the EMSA experiments that reveal additional bands, which can be attributed to the complexes of DNA with two TDP1 molecules (result not shown). Therefore, we suggested that the difference in the regularity of AP site cleavage in each strand can be explained by the antiparallel location of the TDP1 molecules on the DNA strands.

**Figure 3:**
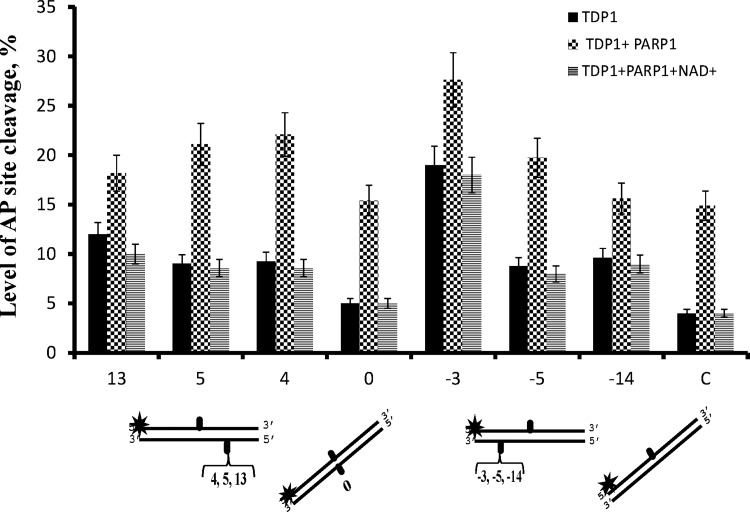
PARP1 stimulation effect depends on the position of the AP site in the opposite strand of the DNA duplex The DNA duplexes (10 nM) containing the AP site in the ^32^P-labelled strand and in defined positions of the opposite strand were treated by TDP1 (10 nM) or TDP1 in the presence of PARP1 (10 nM) or both PARP1 and NAD^+^ (1 mM). Averages and experimental errors were taken from at least three experiments.

**Figure 4:**
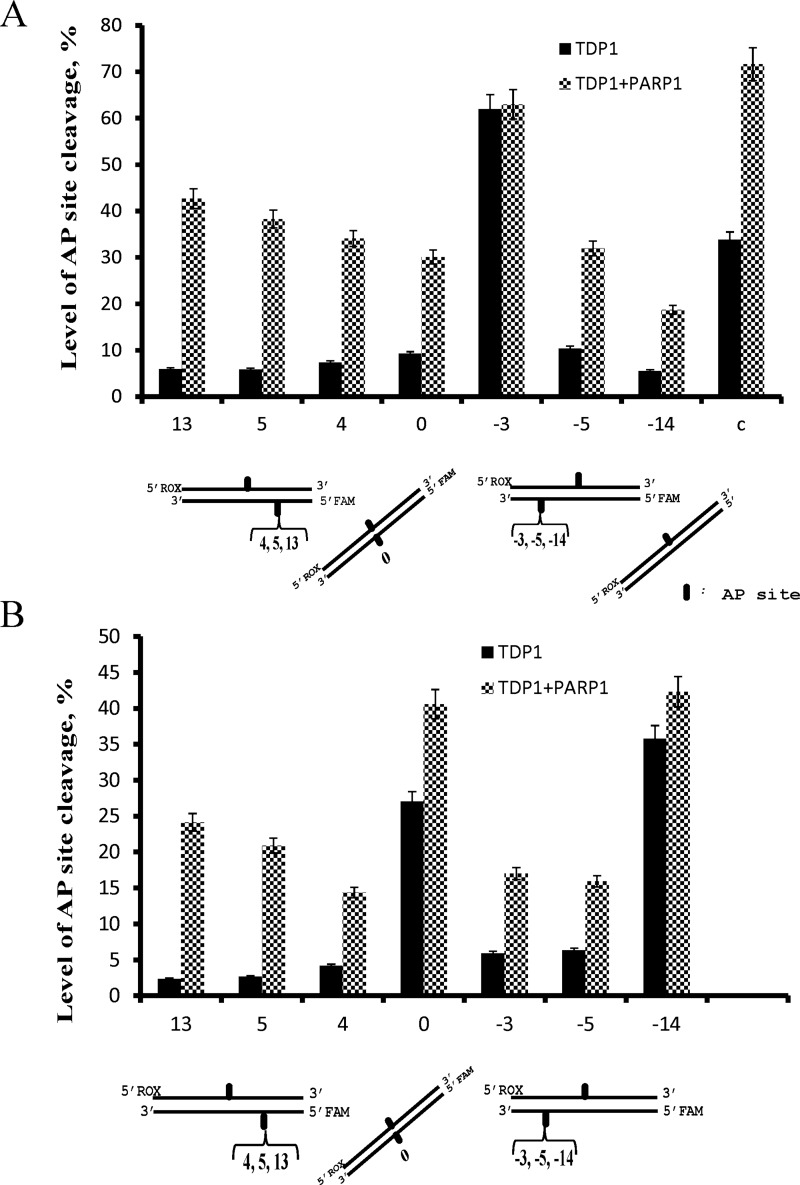
Efficiency of AP site cleavage catalysed by TDP1 in the upper (A) and the bottom strands (B) of the fluorescently-labelled DNA duplexes The fluorescently-labelled DNA duplexes (500 nM) containing the AP site with the FAM in the upper strand and the ROX in the bottom strand were treated with TDP1 (500 nM) or TDP1 in the presence of PARP1 (500 nM). Averages and experimental errors were taken from at least three experiments.

PARP1 was shown to interact with and modulate activity of some BER enzymes [24–26]. As Figure 3 demonstrates, PARP1 simulates the AP-site hydrolysing activity of TDP1. The stimulating effect was more pronounced on the DNA structures that were hydrolysed by TDP1 with initially low efficiency (up to 3.5-fold in the case of duplex DNA with a single AP site). The stimulation effect was abolished by PAR synthesis in the presence of NAD^+^, probably due to dissociation of PARylated PARP1 from the DNA. On the other hand, although TDP1 was recently shown to be a target for PARylation, this modification was considerably less effective in comparison with the PARP1 autoPARylation and did not influence the 3′-tyrosyl-phosphodiesterase activity of TDP1 [18]. It should be noted that PARP1 itself does not cleave the AP site and NAD^+^ alone does not influence the TDP1 activity in the conditions used (result not shown). The stimulation effect of PARP1 was also observed on fluorescently-labelled DNA using higher concentrations of DNA and proteins, but the same ratio of DNA to the proteins (Figure 4). One can observe that PARP1 increases efficiency of the AP site hydrolysis by TDP1 in both DNA strands except for the AP site in the upper strand of the DNA structure −3, which is cleaved effectively by TDP1 alone (Figure 4A). The stimulation effect varies widely, being minimal for the AP site in the bottom strand of the DNA structure −14 (Figure 4B).

**Figure 5:**
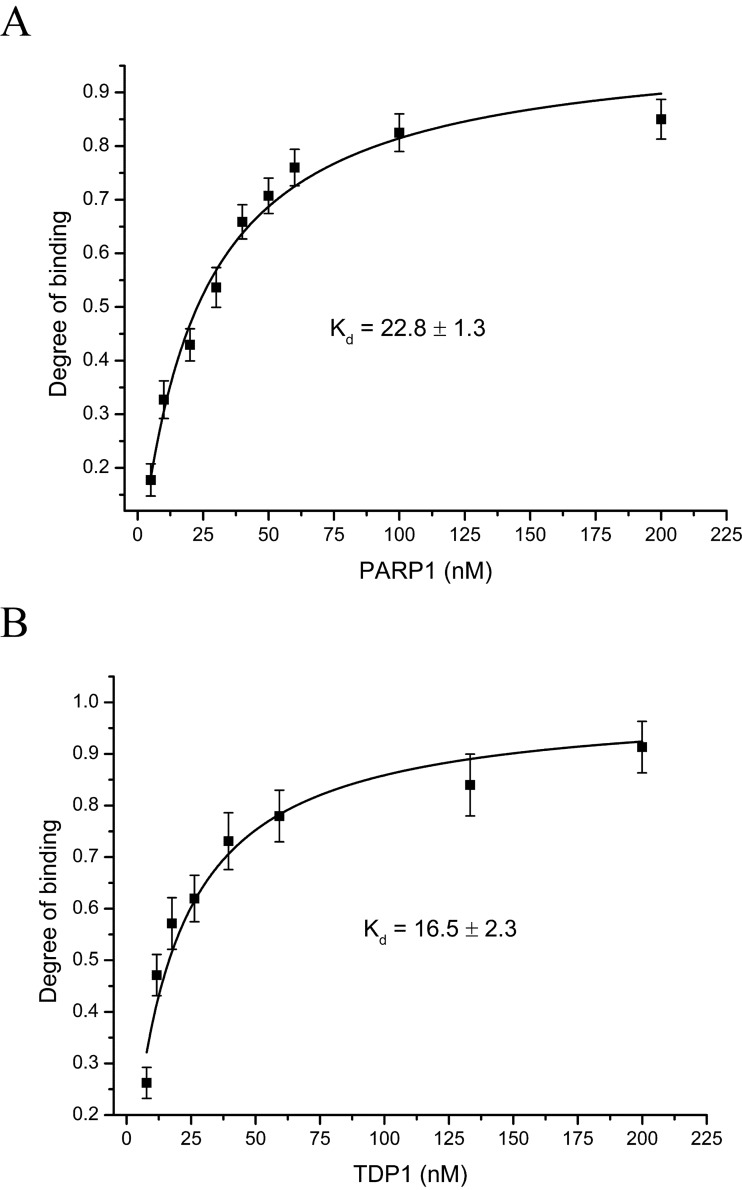
TDP1–PARP1-binding isotherms for fluorescein-labelled TDP1 (A) and PARP1 (B) Fluorescein-labelled protein (20 nM) was titrated with increasing concentrations of the non-labelled protein. Averages and experimental errors were taken from at least three experiments.

Protein–protein interaction between TDP1 and PARP1 seems to be involved in the stimulation of TDP1 activity. To estimate this interaction quantitatively, fluorescently-labelled proteins were used. Figure 5 presents the binding isotherms for fluorescein-labelled TDP1 (A) and PARP1 (B). The dissociation constant values calculated from these titration curves (22.8±1.3 and 16.5±2.3 nM respectively) speak in favour of tight contacts between these proteins.

To further characterize the TDP1–PARP1 interaction, we have utilized FRET measurements. FRET is a spectroscopic process by which a donor fluorophore transfers energy to an acceptor chromophore through non-radiative dipole–dipole coupling. FRET is extremely sensitive to small distances because the efficiency of energy transfer is inversely proportional to the sixth power of the distance between the donor and the acceptor [27]. Therefore, researchers have long been using this photo-physical process to monitor molecular rearrangements in the 1–10 nm range that correlates with the size of the biological macromolecules. Fluorescein and TAMRA dyes are ideal for the FRET process because the fluorescence spectrum of the donor (fluorescein) overlaps with the absorption spectrum of the acceptor (TAMRA).

**Figure 6:**
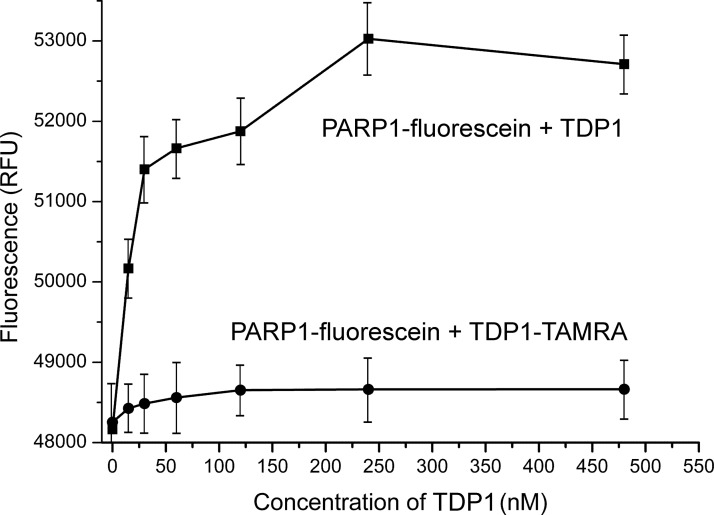
Fluorescence of the PARP1–fluorescein conjugate in the presence of TDP1 or the TDP1–TAMRA conjugates Averages and experimental errors were taken from at least three experiments.

**Figure 7:**
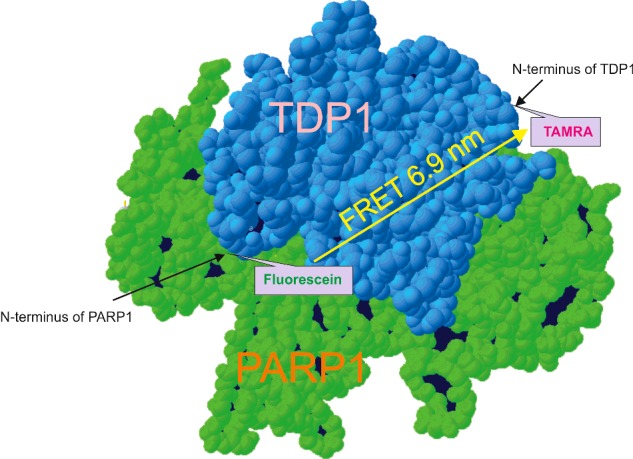
A hypothetical scheme of the antiparallel orientated binding of PARP1 and TDP1 The scheme was created from the N-terminally truncated version of the human TDP1 protein structure (RCSB Protein Data Bank, Residues 149–608, pdb=1jy1 [28]), the reconstructed N-terminal domain of the human TDP1 as unstructured region and the structure of the human PARP1 (domains Zn1, Zn3, WGR–CAT, RCSB Protein Data Bank, pdb=4dqy [29]).

Figure 6 shows that the fluorescence of the PARP1–FAM conjugate decreases when TDP1 is replaced by the TDP1–TAMRA conjugate. The efficiency of energy transfer from the donor to the acceptor (E) can be estimated by:

$$E=\left(1-\frac{I_{D}}{I_{\mathrm{DA}}}\right)\times \frac{1}{A_{l}}$$

where *I*_D_ and *I*_DA_ are the fluorescence intensities of the donor in the absence and in the presence of the acceptor, respectively; *A*_l_ is the degree of TDP1 labelling. The calculated value of *E* is 0.21±0.05. According to the Förster theory on non-radiant energy transfer, the distance (*R*) between the donor and the acceptor can be calculated by:

$$R=R_{0}{\left(\frac{1}{E}-1\right)}^{1/6}$$

where *R_0_* is the Förster distance at which the efficiency of energy transfer is equal to 50%. Using an *R*_0_ of 5.5 nm for the fluorescein–TAMRA pair, we calculated a distance of *R*=6.9 nm between TAMRA at the N-terminus of TDP1 and fluorescein at the N-terminus of PARP1. The distance between the N-termini of TDP1 and PARP1 was not significantly changed upon addition of DNA (from 6.9 to 7.0 nm). On the basis of these data, we present a tentative scheme of TDP1 and PARP1 interaction in Figure 7. PARP1 binds TDP1 in an antiparallel orientation; the N-terminus of the former protein interacts with the C-terminal domain of the latter protein.

Thus, we reveal a physical interaction of PARP1 with TDP1 independent of DNA damage and PARylation. The antiparallel orientation of PARP1 and TDP1 promotes the functional interaction of both proteins. Our data on the mode of the protein–protein interaction are in agreement with the data reported in the previously-mentioned article [18]. Using pull-down analysis, the authors showed that the N-terminal domain of TDP1 directly binds to the C-terminal domain of PARP1. Hence, we suggest that due to protein–protein interaction, PARP1 stimulates the AP-site cleavage activity of TDP1. Therefore, our data show for the first time the functional significance of the PARP1 and TDP1 interaction in the process of DNA repair. The detailed mechanism of the stimulation of the AP-site cleavage activity of TDP1 by PARP1 requires further analyses.

## Abbreviations

AP site: apurinic/apyrimidinic site
BER: base excision repair
dUMP: deoxyuridine monophosphate
FAM: 5′-fluorescein
NHS: N-hydroxysuccinimide
PAR: poly(ADP-ribose)
PARP1: poly(ADP-ribose)polymerase 1
ROX: 5′-rhodamine
TAMRA: tetramethyl-6-carboxyrhodamine
TDP1: tyrosyl–DNA phosphodiesterase 1
UDG: uracil–DNA glycosylase

## References

[1] Sung J.S., Demple B. (2006). Roles of base excision repair subpathways in correcting oxidized abasic sites in DNA. FEBS J..

[2] Gulston M., de Lara C., Jenner T., Davis E., O’Neill P. (2004). Processing of clustered DNA damage generates additional double-strand breaks in mammalian cells post-irradiation. Nucleic Acids Res..

[3] Lindahl T., Satoh M.S., Poirier G.G., Klungland A. (1995). Post-translational modification of poly(ADP-ribose) polymerase induced by DNA strand breaks. Trends Biochem. Sci..

[4] Khodyreva S.N., Ilina E.S., Sukhanova M.V., Kutuzov M.M., Lavrik O.I. (2010). Poly(ADP-ribose) polymerase 1 interaction with apurinic/apyrimidinic sites. Dokl. Akad. Nauk..

[5] Khodyreva S.N., Prasad R., Ilina E.S., Sukhanova M.V., Kutuzov M.M., Liu Y., Hou E.W., Wilson S.H., Lavrik O.I. (2010). Apurinic/apyrimidinic (AP) site recognition by the 5’-dRP/AP lyase in poly(ADP-ribose) polymerase-1 (PARP-1). Proc. Natl. Acad. Sci. U.S.A..

[6] Lebedeva N.A., Rechkunova N.I., Lavrik O.I. (2011). AP-site cleavage activity of tyrosyl-DNA phosphodiesterase 1. FEBS Lett..

[7] Lebedeva N.A., Rechkunova N.I., El-Khamisy S.F., Lavrik O.I. (2012). Tyrosyl-DNA phosphodiesterase 1 initiates repair of apurinic/apyrimidinic sites. Biochimie.

[8] Lebedeva N.A., Rechkunova N.I., Ishchenko A.A., Saparbaev M., Lavrik O.I. (2013). The mechanism of human tyrosyl-DNA phosphodiesterase 1 in the cleavage of AP site and its synthetic analogs. DNA Repair.

[9] Lebedeva N.A., Rechkunova N.I., Lavrik O.I. (2014). Repair of apurinic/apyrimidinic sites in single-stranded DNA initiated by tyrosyl-DNA phosphodiesterase 1. Dokl. Biochem. Biophys..

[10] Yang S.W., Burgin A.B., Huizenga B.N., Robertson C.A., Yao K.C., Nash H.A. (1996). A eukaryotic enzyme that can disjoin dead-end covalent complexes between DNA and type I topoisomerases. Proc. Natl. Acad. Sci. U.S.A..

[11] Pouliot J.J., Yao K.C., Robertson C.A., Nash H.A. (1999). Yeast gene for a Tyr-DNA phosphodiesterase that repairs topoisomerase I complexes. Science.

[12] Interthal H., Pouliot J.J., Champoux J.J. (2001). The tyrosyl-DNA phosphodiesterase TDP1 is a member of the phospholipase D superfamily. Proc. Natl. Acad. Sci. U.S.A..

[13] Rass U., Ahel I., West S.C. (2007). Defective DNA repair and neurodegenerative disease. Cell.

[14] Interthal H., Chen H.J., Champoux J.J. (2005). Human TDP1 cleaves a broad spectrum of substrates, including phosphoamide linkages. J. Biol. Chem..

[15] Zhou T., Lee J.W., Tatavarthi H., Lupski J.R., Valerie K., Povirk L.F. (2005). Deficiency in 3’-phosphoglycolate processing in human cells with a hereditary mutation in tyrosyl-DNA phosphodiesterase (TDP1). Nucleic Acids. Res..

[16] Dexheimer T.S., Antony S., Marchand C., Pommier Y. (2008). Tyrosyl-DNA phosphodiesterase as a target for anticancer therapy. Anticancer Agents Med. Chem..

[17] Prasad R., Williams J.G., Hou E.W., Wilson S.H. (2012). Pol β associated complex and base excision repair factors in mouse fibroblasts. Nucleic Acids Res..

[18] Das B.B., Huang S.Y., Murai J., Rehman I., Amé J.C., Sengupta S., Das S.K., Majumdar P., Zhang H., Biard D. (2014). PARP1-TDP1 coupling for the repair of topoisomerase I-induced DNA damage. Nucleic Acids Res..

[19] El-Khamisy S.F., Saifi G.M., Weinfeld M., Johansson F., Helleday T., Lupski J.R., Caldecott K.W. (2005). Defective DNA single-strand break repair in spinocerebellar ataxia with axonal neuropathy-1. Nature.

[20] Sukhanova M.V., Khodyreva S.N., Lavrik O.I. (2004). Poly(ADP-ribose) polymerase-1 inhibits strand-displacement synthesis of DNA catalyzed by DNA polymerase beta. Biochemistry.

[21] Sambrook J., Fritsch E.F., Maniatis T. (1989). Molecular Cloning: A Laboratory Manual.

[22] Haugland R.P. (2005). The Handbook–a Guide to Fluorescent Probes and Labeling Technologies.

[23] Constant J.F., Demeunynck M., Demeunynck M., Baily C., Wilson W.D. (2003). Design and studies of abasic site targeting drugs: new strategies for cancer chemotherapy. In Small Molecule DNA and RNA Binders. From Synthesis to Nucleic Acid Complexes.

[24] Prasad R., Lavrik O.I., Kim S.J., Kedar P., Yang X.P., Berg B.J., Wilson S.H. (2001). DNA polymerase beta-mediated long patch base excision repair. Poly(ADP-ribose)polymerase-1 stimulates strand displacement DNA synthesis. J. Biol. Chem..

[25] Cistulli C., Lavrik O.I., Prasad R., Hou E., Wilson S.H. (2004). AP endonuclease and poly(ADP-ribose) polymerase-1 interact with the same base excision repair intermediate. DNA Repair.

[26] Sukhanova M.V., Khodyreva S.N., Lebedeva N.A., Prasad R., Wilson S.H., Lavrik O.I. (2005). Human base excision repair enzymes apurinic/apyrimidinic endonuclease1 (APE1), DNA polymerase beta and poly(ADP-ribose) polymerase 1: interplay between strand-displacement DNA synthesis and proofreading exonuclease activity. Nucleic Acids Res..

[27] Förster T. (1948). Intermolecular energy migration and fluorescence. Ann. Phys..

[28] Davies D.R., Interthal H., Champoux J.J., Hol W.G. (2002). The crystal structure of human tyrosyl-DNA phosphodiesterase, Tdp1. Structure.

[29] Langelier M.F., Planck J.L., Roy S., Pascal J.M. (2012). Structural basis for DNA damage-dependent poly(ADP-ribosyl)ation by human PARP-1. Science.

